# The Goodyear Patent

**Published:** 1881-09

**Authors:** 


### THE GOODYEAR PATENT.

Our readers will be pleased to be assured that the Goodyear Company do not intend to make an attempt to extend their patents on dental vulcanite. This we state authoritatively, having had a communication from them to that effect. For months past speculation and conjectures have been afloat as to whether or not the Company intended to make another attempt to clutch the dental profession. The fact that no move on their part was discernable appeared to justify us in coming to the conclusion that they had decided to allow the patent to lapse. But, on the other hand, dentists thought that such a course was hardly possible. The action of the Company in the past had been so grasping that it seemed to be contrary to their nature to allow even the possibility of success in a renewal of their patent to pass them without seizing at it. Their last step, however, has been better than we judged.—*Johnston's Dental Miscellany.*

